# Association of Country-Specific Socioeconomic Factors With Survival of Patients Who Experience Severe Classic Acute Graft-vs.-Host Disease After Allogeneic Hematopoietic Cell Transplantation. An Analysis From the Transplant Complications Working Party of the EBMT

**DOI:** 10.3389/fimmu.2020.01537

**Published:** 2020-07-23

**Authors:** Andrzej Frankiewicz, Christophe Peczynski, Sebastian Giebel, Alenca Harrington, Gerard Socié, Dietger Niederwieser, Christoph Scheid, Martin Bornhäuser, Nicolaus Kröger, Ahmet Elmaagacli, Boris Afanasyev, Peter Dreger, Claudia Rössig, Didier Blaise, Christian Kratz, Ibrahim Yakoub-Agha, Bernhard Kremens, Charlotte Marie Niemeyer, Gerald Wulf, Igor Blau, Olaf Penack, Hildegard Greinix, Grzegorz W. Basak

**Affiliations:** ^1^The Department of Bone Marrow Transplantation and Onco-Hematology, Maria Sklodowska-Curie National Research Institute of Oncology, Gliwice, Poland; ^2^EBMT Paris Study Office/CEREST-TC Hôpital Saint Antoine, Paris, France; ^3^Hopital St. Louis, Department of Hematology – BMT 1, Paris, France; ^4^Division of Hematology, Oncology and Hemostasiology, University Hospital Leipzig, Leipzig, Germany; ^5^I. Department of Medicine, University of Cologne, Cologne, Germany; ^6^Universitaetsklinikum Dresden Medizinische Klinik und Poliklinik, Dresden, Germany; ^7^Department of Stem Cell Transplantation, University Hospital Eppendorf, Hamburg, Germany; ^8^Department of Haematology, Asklepios Klinik St. Georg, Hamburg, Germany; ^9^Raisa Gorbacheva Memorial Research Institute for Paediatric Oncology, Hematology, and Transplantation, First State Pavlov Medical University of St. Petersburg, St. Petersburg, Russia; ^10^University of Heidelberg Medizinische Klinik und Poliklinik, Heidelberg, Germany; ^11^Universitaetsklinikum Muenster Klinik für Kinder- und Jugendmedizin - Pädiatrische Hämatologie und Onkologie, Münster, Germany; ^12^Programme de Transplantation & Therapie Cellulaire Centre de Recherche en Cancérologie de Marseille Institut Paoli Calmettes, Marseille, France; ^13^Department of Pediatric Haematology/Oncology, Hannover Medical University, Hanover, Germany; ^14^Hôpital HURIEZ UAM allo-CSH CHRU, Lille, France; ^15^Department of Pediatric Hematology/Oncology, University Hospital, Essen, Germany; ^16^Pediatric Hematology and Oncology, Department of Pediatrics and Adolescent Medicine, University Medical Centre, Freiburg, Germany; ^17^Universitaetsklinikum Goettingen Abteilung Hämatologie und Onkologie, Gottingen, Germany; ^18^Department of Hematology, Oncology and Tumorimmunology, Charite Universitätsmedizin Berlin, Berlin, Germany; ^19^Klinische Abteilung für Hämatologie, Universitätsklinik für Innere Medizin, Graz, Austria; ^20^Department of Haematology, Oncology and Internal Medicine, Medical Uniwersity of Warsaw, Warsaw, Poland

**Keywords:** health care expenditure, human development index, hematopoietic cell transplantation, acute graft-vs.-host disease, transplant-related mortality

## Abstract

Acute graft-vs.-host disease (aGvHD) is one of the most frequent causes of transplant-related mortality (TRM) after allogeneic hematopoietic cell transplantation (alloHCT). Its treatment is complex and costly. The aim of this study was to retrospectively analyze the impact of country-specific socioeconomic factors on outcome of patients who experience severe aGvHD. Adults with hematological malignancies receiving alloHCT from either HLA-matched siblings (*n* = 1,328) or unrelated donors (*n* = 2,824) developing grade 3 or 4 aGvHD were included. In univariate analysis, the probability of TRM at 2 years was increased for countries with lower current Health Care Expenditure (HCE, *p* = 0.04), lower HCE as % of Gross Domestic Product per capita (*p* = 0.003) and lower values of the Human Development Index (*p* = 0.02). In a multivariate model, the risk of TRM was most strongly predicted by current HCE (HR = 0.76, *p* = 0.006). HCE >median was also associated with reduced risk of the overall mortality (HR 0.73, *p* = 0.0006) and reduced risk of treatment failure (either relapse or TRM; HR 0.77, *p* = 0.004). We conclude that country-specific socioeconomic factors, in particular current HCE, are strongly associated with survival of patients who experience severe aGvHD.

## Introduction

Allogeneic hematopoietic cell transplantation (alloHCT) is widely used for the treatment of hematological malignancies. Unfortunately, despite improvements that have been made, it is still associated with significant morbidity and mortality limiting its clinical benefits (1). Acute graft-vs.-host disease (aGvHD) is one of the most frequent complications of alloHCT. Its severe form occurs in 14% of transplant recipients being one of the most frequent causes of transplant-related mortality (TRM) (2, 3). Initial treatment of aGvHD is well established. It consists of high doses of glucocorticosteroids and is effective in approximately two thirds of the patients (4). Treatment of steroid-refractory aGvHD is not well standardized and is associated with dismal prognosis (5). Furthermore, it must be accompanied by intensive supportive care to treat secondary complications, in particular infections associated with profound immunosuppression. Altogether, the therapy of severe aGvHD is complex and costly. It requires experience, but also resources and appropriate settings.

It may be hypothesized that factors other than direct medical care during initial hospital stay contribute to short - and long-term survival of patients who experience severe aGvHD. The recovery may be long-lasting requiring intense social support and easy access to basic as well as specialized medical services in the area of residence. Living conditions may also influence outcome. In view of these considerations the prognosis of the patients may vary between countries and depend on many factors including their socioeconomic status, organization of the transplant program at a national level and finally—on individual team experience. Association of these factors with survival after alloHCT was a subject of several studies run by the European Society for Blood and Marrow Transplantation (EBMT) (6–9). However, these associations have not been explored so far with regard to particular transplant-related complications. Hence, the aim of this study was to analyze the potential influence of macroeconomic and socioeconomic factors together with team density per population and per country area as well as team activity on long-term outcome of patients who experience severe aGvHD.

## Patients and Methods

### Study Design and Data Collection

This was a retrospective, multicenter analysis based on data provided by the EBMT registry for transplants performed between the years 2011 and 2015. Centers participating in the EBMT are annually requested to report all consecutive transplantation procedures and patients' follow-up. The validation and quality control program includes verification of the computer print-out of the entered data, cross-checking with the national registries, and annually on-site visits of selected teams.

Data on HCE as well as data on country areas and populations were obtained from the Eurostat (http://appsso.eurostat.ec.europa.eu) for the year 2013. For calculation of team density per population and per area, the number of transplant teams was counted based on the EBMT membership. HDI values for the year 2013 were obtained from the Human Development Report 2014 published by the United Nations (http://hdr.undp.org/sites/default/files/hdr14-report-en-1.pdf). The values are presented in Table 1.

**Table 1:** Patients and donors, transplantation procedure, economic, and socioeconomic indices.

**Patient and procedure characteristics**	
*N*	4,152
Median patient age, years (range)	53 (18–79)
Median donor age, years (range)	37 (5–77)
*Missing data*	*2,344*
Median year of transplantation (range)	2,013 (2,011–2,015)
Median interval from diagnosis to transplantation, range (days)	296 (7–13,058)
**Donor type**	
HLA-identical sibling	1,328 (32%)
Unrelated	2,824 (68%)
HLA-matched	1,491 (72%)
HLA-mismatched	576 (28%)
*Missing data*	*757*
**Donor/recipient gender**	
Female to male	851 (21%)
Other combination	3,255 (79%)
*Missing data*	*46*
**Donor/recipient CMV serological status**	
Negative/negative	1,089 (27.5%)
Other	2,871 (72.5%)
*Missing data*	*192*
**Diagnosis**	
Acute leukemia	2,091 (50%)
Chronic leukemia	432 (10%)
Lymphoma	560 (14%)
Plasma cell disorders	185 (5%)
MDS/MPN	884 (21%)
Disease risk status at alloHCT^*^	
High risk	1,387 (35%)
Low/moderate risk	2,625 (65%)
*Missing*	*140*
**Previous autoHCT**	
No	3,645 (88%)
Yes	507 (12%)
**Source of stem cells**	
Bone marrow	356 (9%)
Peripheral blood	3,796 (91%)
***In vivo*** **T-cell depletion**	
No	1,870 (45.7%)
Yes	2,223 (54.3%)
**Conditioning intensity**	
Myeloablative	2,047 (49%)
Reduced intensity	2,096 (51%)
*Missing data*	*9*
**Type of conditioning**	
Chemotherapy-based	3,027 (73%)
Total body irradiation-based	1,122 (27%)
*Missing data*	*3*
**Economic and socioeconomic indices**	**Median (range)**
Current HCE, range (Euro)	4,955 (465–9,720)
Median HCE as % of GDP	11.1 (3.7–12)
Median private HCE, range (Euro)	1,087 (103–3,168)
Median public HCE, range (Euro)	3,868 (308–8,285)
Median no. teams per 1 million inhabitants	1.09 (0.03–1.62)
Median no. teams per 1,000 km^2^	0.20 (0.00–0.59)
Median HDI	0.88 (0.74–0.94)
Median no. alloHCT^**^ between 2004 and 2008	13.5 (2.0–35.0)

*MDS indicates myelodysplastic syndrome; MPN, myeloproliferative neoplasms; HCE health care expenditure; HDI, human development index; GDP, gross domestic product per capita*.

* *For the purpose of this study the definition of high risk related to underlying disease was defined as follows: acute leukemias and lymphomas with active disease, chronic myeloid leukemia in acceleration phase or in blast crisis, myelodysplastic syndromes and myeloproliferative neoplasms not responding to preceding therapy*.

** *Refers to transplantation procedures according to selection criteria chosen for this study*.

The study was approved by the Transplant Complications Working Party of the EBMT institutional review board and conducted in accordance with the Declaration of Helsinki and Good Clinical Practice guidelines. Since 1990, all patients provide informed consent authorizing the use of their personal information for research purposes.

### Criteria of Selection

Inclusion criteria were as follows: (1) the diagnosis of hematological malignancy, (2) age ≥18 years, (3) alloHCT from either HLA-matched sibling (MSD) or unrelated donor (URD), (4) alloHCT performed between 2011 and 2015 in European centers reporting to the EBMT registry, (5) bone marrow or peripheral blood used as a source of stem cells, (6) evidence of “classic” grade III or IV aGvHD according to Glucksberg/Seattle criteria, developing until day 100 after alloHCT (10).

### Statistical Analysis

The probability of TRM was the primary study end-point. Overall survival (OS), progression incidence (PI), progression-free survival (PFS), and the incidence of overall and extensive chronic graft-vs.-host disease (cGvHD) were secondary study end-points. In the analysis of causes of mortality, GvHD was reported as cause of death if the patient died before resolution of GvHD symptoms.

In univariate analyses, TRM and PI were calculated using cumulative incidence curves in a competing risks setting, death in remission being treated as a competing event to progression (11). For the calculation of cGvHD incidence, both TRM and progression represented competing risks. The PFS was defined as time interval from alloHCT to either relapse or death in remission while OS was time from alloHCT to death from any cause. The comparisons were done with the use of the Gray test for NRM, PI and cGvHD, and log-rank test for OS and PFS.

HCE (current, public, private, and as % of GDP), team density per country population and area, HDI, and center experience (no. of alloHCT meeting selection criteria for this study, performed in a study period) were independent variables. For the purpose of the analyses they were categorized by medians. For current HCE and public HCE the lists of countries after categorization were exactly the same, hence, public HCE could not be analyzed separately.

In multivariate analyses each socioeconomic factor, one by one, was added to a Cox's cause-specific proportional hazard model including other potential risk factors (recipient age, donor type, donor/recipient gender and cytomegalovirus serological status, diagnosis, interval from diagnosis to alloHCT, disease risk status, year of transplantation, source of stem cells, conditioning intensity, use of *in vivo* T-cell depletion, use of TBI). In order to take non-independence of data within a country into account, a random effect or frailty was introduced for each country into the models (12, 13). A frailty is a latent random effect that enters multiplicatively on the hazard function.

The median follow-up for survivors was 29 months. All tests were two-sided with type I error rate fixed at 0.05. Statistical analyses were performed with SPSS 24.0 (IBM Corp., Armonk, NY, USA) and R 3.4.2 (R Core Team (2017). R: A language and environment for statistical computing. R Foundation for Statistical Computing, Vienna, Austria. URL https://www.R-project.org/).

## Results

### Patients, Donors, and HCT Procedure

Altogether 4,152 patients, including 2,573 men, treated with alloHCT from either MSD (*n* = 1,328, 32%) or URD (*n* = 2,824, 68%) in 282 transplant centers located in 30 European countries were included in the analysis. Median age was 53 years (range, 18–79 years). Acute leukemias were the most frequent indication for transplantation (*n* = 2,091, 50%), followed by myelodysplastic syndromes and myeloproliferative neoplasms (*n* = 884, 21%). Peripheral blood was the predominant source of stem cells (*n* = 3,796, 91%). The conditioning regimens were myeloablative or reduced intensity, in almost equal proportions. Detailed patients and procedure characteristics are listed in Table 1.

### Transplant-Related Mortality

The TRM rates at 6, 12, and 24 months for the whole group were 41% (95%CI: 39–42), 51% (49–52), and 56% (54–57), respectively. The most frequent causes of TRM were: GvHD (74.8%), infections (19%), veno-occlusive disease (2.3%), interstitial pneumonitis (1.2%), and hemorrhage (1%). However, in 223 out of 2,324 (5.3%) cases the cause of death was reported as other or unknown.

**Figure 1:**
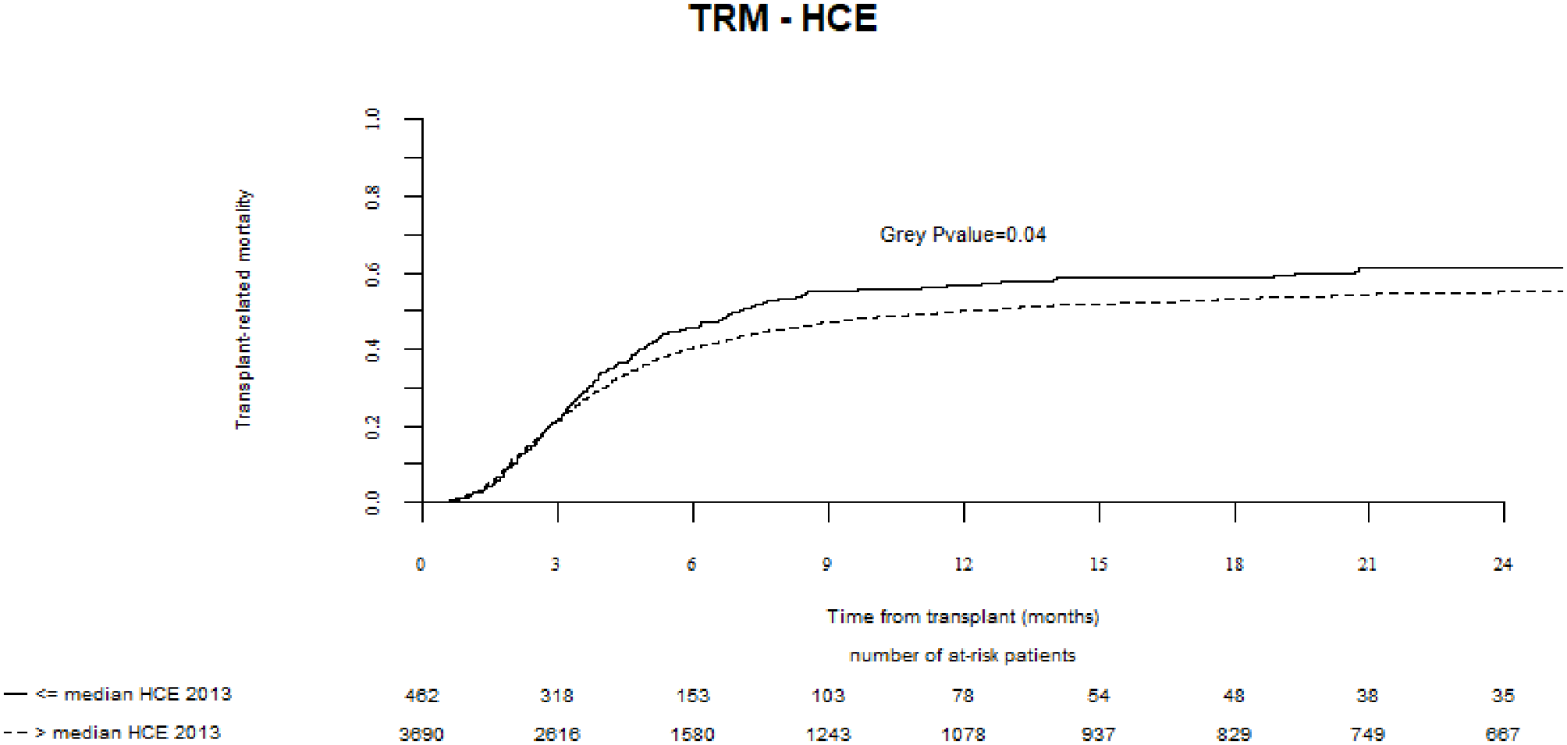
Association of current Health Care Expenditure with transplant related mortality.

In a univariate analysis, the probability of TRM at 2 years was increased for countries with lower current HCE (≤median vs. >median, 61 vs. 55%, respectively, *p* = 0.04), lower HCE as % of Gross Domestic Product (GDP) *per capita* (60 vs. 54%, *p* = 0.003), and lower values of HDI (59 vs. 55%, *p* = 0.02) (Table 2, Figure 1). In a multivariate analysis, the strongest effect was observed where current HCE was included in the model (hazard ratio, HR = 0.76, 95%CI, 0.62–0.92; *p* = 0.006). Significant associations were also observed for models including HCE as % of GDP and HDI (Table 3). No significant associations were found between TRM and team density or individual team activity.

**Table 2:** Results of the univariate analysis of associations of economic and socioeconomic factors with outcome.

		**TRM (2y) (%, 95%CI)**	**OS (2y) (%, 95%CI)**	**PI (2y) (%, 95%CI)**	**PFS (2y) (%, 95%CI)**	**Overall cGvHD (2y) (%, 95%CI)**	**Extensive cGvHD (2y) (%, 95%CI)**
HCE current	≤ median	61 (56–66)	23 (18–28)	17 (13–22)	22 (17–27)	27 (21–32)	17 (13–22)
	> median	55 (53–57)	32 (30–33)	16 (14–17)	29 (28–31)	37 (35–39)	24 (22–25)
	*p*-value	0.04	0.002	0.69	0.013	0.002	0.01
HCE as % of GDP	≤ median	60 (56–63)	25 (22–29)	17 (15–20)	23 (20–27)	27 (23–30)	18 (15–21)
	> median	54 (53–56)	32 (31–34)	15 (14–17)	30 (29–32)	39 (37–40)	24 (23–26)
	*p*-value	0.003	<0.001	0.17	<0.001	<0.001	<0.001
HCE private	≤ median	58 (54–62)	27 (24–31)	15 (13–18)	26 (23–30)	35 (31–39)	21 (18–25)
	> median	55 (53–57)	32 (30–33)	16 (15–17)	29 (28–31)	36 (35–38)	23 (22–25)
	*p*-value	0.51	0.26	0.73	0.76	1.0	0.25
HDI	≤ median	59 (55–63)	25 (22–29)	18 (15–21)	23 (19–27)	27 (23–31)	17 (14–21)
	> median	55 (53–56)	32 (31–34)	15 (14–17)	30 (28–32)	38 (36–40)	24 (22–26)
	*p*-value	0.019	<0.001	0.31	0.001	<0.001	<0.001
Team density per population	≤ median	58 (54–63)	28 (23–32)	15 (12–18)	27 (23–31)	37 (32–42)	23 (18–27)
	> median	55 (53–57)	31 (30–33)	16 (15–17)	29 (27–31)	36 (34–38)	23 (21–25)
	*p*-value	0.33	0.14	0.85	0.4	0.70	0.38
Team density per area	≤ median	55 (50–60)	31 (27–35)	16 (13–19)	29 (25–34)	38 (33–43)	24 (19–28)
	> median	56 (54–57)	31 (29–33)	16 (14–17)	29 (27–30)	36 (34–38)	23 (21–24)
	*p*-value	0.72	0.93	0.96	0.67	0.62	0.44
No. alloHCT^*^ (team activity)	≤ median	58 (55–61)	29 (26–32)	14 (12–16)	28 (25–31)	37 (34–41)	23 (20–26)
	> median	55 (53–57)	32 (30–34)	16 (15–18)	29 (27–31)	36 (34–38)	23 (21–25)
	*p*-value	0.04	0.13	0.06	0.58	0.12	0.77

*HCE indicates health care expenditure; GDP, gross domestic product per capita; HDI, human development index; TRM, transplant-related mortality; OS, overall survival; PI, progression incidence; PFS, progression-free survival; cGvHD, chronic graft-vs.-host disease; CI, confidence interval*.

* *Refers to transplantation procedures according to selection criteria chosen for this study preformed between 2011 and 2015*.

**Table 3:** Results of the multivariate analysis of associations of economic and socioeconomic factors with transplant-related mortality.

	**TRM**		**OS**		**PI**	
**Factor**	**HR (95%CI)**	***p*-value**	**HR (95%CI)**	***p*-value**	**HR (95%CI)**	***p*-value**
HCE current	0.76 (0.63–0.93)	0.006	0.74 (0.62–0.88)	0.0007	0.89 (0.63–1.25)	0.49
HCE as % of GDP	0.80 (0.67–0.95)	0.01	0.77 (0.66–0.89)	0.0004	0.74 (0.57–0.97)	0.03
HCE private	0.83 (0.68–1.02)	0.08	0.80 (0.66–0.97)	0.02	0.93 (0.69–1.26)	0.65
Team density per population	0.90 (0.75–1.08)	0.25	0.87 (0.73–1.03)	0.10	0.99 (0.72–1.35)	0.93
Team density per area	1.01 (0.83–1.22)	0.92	0.98 (0.82–1.17)	0.81	1.05 (0.77–1.43)	0.75
HDI	0.80 (0.66–0.96)	0.017	0.77 (0.65–0.90)	0.002	0.81 (0.60–1.08)	0.15
No. alloHCT^*^ (team activity)	0.93 (0.74–1.17)	0.54	0.92 (0.75–1.13)	0.42	1.08 (0.83–1.42)	0.57
	**PFS**		**Overall cGvHD**		**Extensive cGvHD**	
	**HR (95%CI)**	***p*****-value**	**HR (95%CI)**	***p*****-value**	**HR (95%CI)**	***p*****-value**
HCE current	0.79 (0.66–0.94)	0.007	1.20 (0.89–1.61)	0.24	1.16 (0.85–1.58)	0.36
HCE as % of GDP	0.78 (0.68–0.9)	0.0006	1.27 (1.06–1.52)	0.009	1.17 (0.94–1.45)	0.17
HCE private	0.85 (0.71–1.02)	0.09	1.09 (0.82–1.46)	0.54	1.13 (0.91–1.39)	0.26
Team density per population	0.91 (0.77–1.08)	0.27	0.82 (0.67–1.01)	0.06	0.83 (0.66–1.04)	0.11
Team density per area	1.01 (0.85–1.20)	0.90	0.85 (0.66–1.08)	0.18	0.87 (0.70–1.09)	0.22
HDI	0.80 (0.68–0.94)	0.007	1.26 (1.04–1.52)	0.02	1.25 (0.99–1.59)	0.06
No. alloHCT^*^ (team activity)	0.91 (0.77–1.08)	0.27	0.90 (0.77–1.05)	0.17	1.02 (0.86–1.20)	0.84

* *Refers to transplantation procedures according to selection criteria chosen for this study performed between 2011 and 2015*.

### Progression Incidence

The PI at 2 years for the whole group was 16% (95%CI: 15–17).

In a univariate analysis the economic and socioeconomic factors had no significant influence on the RI (Table 2). However, in a multivariate model adjusted for other prognostic factors, the risk of progression was significantly decreased for centers located in countries with HCE as % of GDP >median (HR = 0.74, 95%CI, 0.57–0.97, *p* = 0.03) (Table 3).

### Progression-Free Survival and Overall Survival

The PFS and OS rates at 2 years were 29% (95%CI: 27–30) and 31% (95%CI: 29–33), respectively. Among 2,691 patients who died, the causes of death were assessed as transplant-related in 2,090 (78%) cases, disease-related in 408 (15%) cases while other or unknown in remaining 194 (7%) cases.

**Figure 2:**
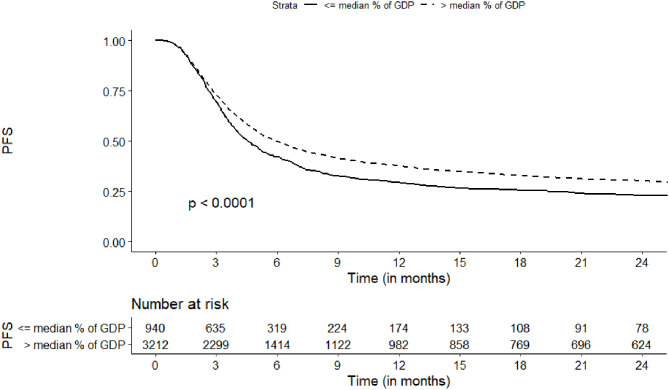
Association of Health Care Expenditure as % of Gross Domestic Product per Capita with progression free survival.

In a univariate analysis, the probability of PFS at 2 years was increased for countries with higher current HCE (>median vs. ≤median, 29 vs. 22%, respectively, *p* = 0.01), higher HCE as % of Gross Domestic Product (GDP) *per capita* (30 vs. 23%, *p* < 0.001), and higher values of HDI (30 vs. 23%, *p* = 0.001) (Table 2, Figure 2). The same factors were identified to influence the risk of treatment failure (either progression or death without progression, inverse PFS) in a multivariate model (Table 3). The strongest association was found for HCE as % of GDP (HR = 0.78, 95%CI, 0.68–0.90; *p* = 0.0006).

**Figure 3:**
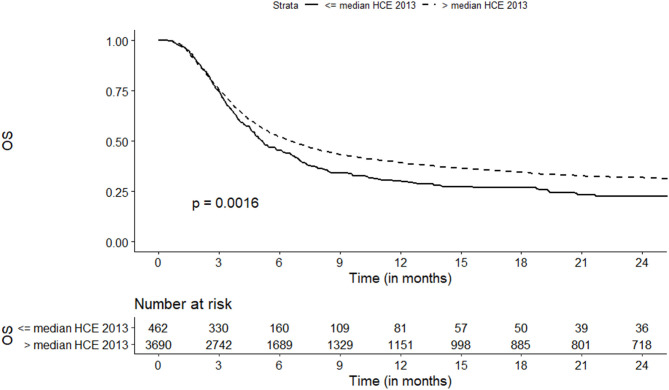
Association of Health Care Expenditure current with overall survival.

**Figure 4:**
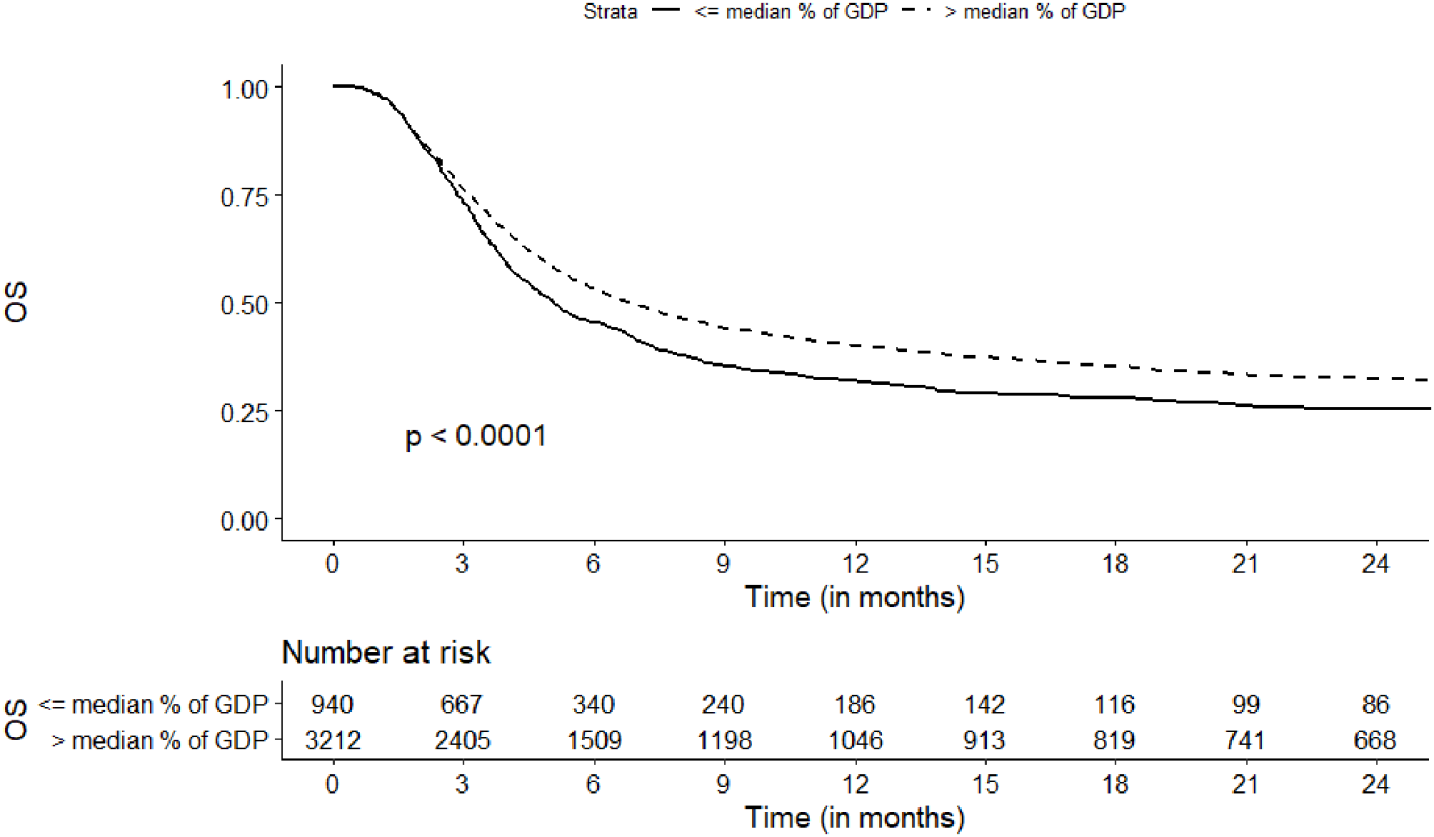
Association of Health Care Expenditure as % of Gross Domestic Product per Capita with overall survival.

The probability of OS at 2 years was increased for centers located in countries with higher current HCE (32 vs. 23%, *p* = 0.002) and higher HDI (32 vs. 25%, *p* < 0.001) (Table 2, Figure 3). In a multivariate analysis the strongest association was observed where current HCE was included in the model (HR = 0.74, 95%CI, 0.62–0.88; *p* = 0.0007). The risk of overall mortality was also decreased for countries with higher HCE as % of GDP (Figure 4), higher private HCE and higher values of HDI (Table 3).

No significant associations between team density or team experience and PFS or OS could be demonstrated.

In a model including current HCE, the risk of both TRM and overall mortality was also affected by recipient age, donor type, donor/recipient CMV serological status, conditioning intensity and disease risk status (Table 4).

**Table 4:** A multivariate analysis of factors associated with transplant-related mortality and overall survival.

	**TRM**		**OS**	
**Factor**	**HR (95%CI)**	***p*-value**	**HR (95%CI)**	***p*-value**
Current HCE (> median vs. ≤ median)	0.76 (0.63–0.93)	0.006	0.74 (0.62–0.88)	0.0007
Patient age (10-year increase)	1.22 (1.17–1.27)	<0.0001	1.2 (1.16–1.25)	<0.0001
Donor/patient CMV serological status (neg/neg vs. other combinations)	0.84 (0.76–0.94)	0.002	0.87 (0.79–0.96)	0.004
Disease Risk status (high vs. low)	1.18 (1.07–1.31)	0.001	1.4 (1.28–1.53)	<0.0001
Donor type (unrelated vs. identical sibling)	1.35 (1.21–1.51)	<0.0001	1.28 (1.16–1.41)	<0.0001
Conditioning intensity (reduced vs. myeloablative)	0.82 (0.74–0.91)	0.0002	0.86 (0.78–0.94)	0.001
Country (frailty)		0.0009		0.0007

*HCE indicates health care expenditure*.

### Chronic GVHD

The cumulative incidence of overall cGvHD and extensive cGvHD at 2 years for the whole group was 36% (95%CI: 35–38) and 23% (95%CI: 21–24), respectively.

The incidence of overall cGvHD was increased for countries with higher current HCE (37 vs. 27%, *p* = 0.002), higher HCE as % of GDP (39 vs. 27%, *p* < 0.001), and higher HDI (38 vs. 27%, *p* < 0.001) (Table 2). In multivariate models the risk of overall cGvHD was increased for countries with higher HCE as % of GDP (Table 3).

In a univariate analysis the incidence of extensive cGvHD was increased for countries with current HCE, HCE as % of GDP and HDI higher than median (Table 2). These associations, however, lost their statistical significance after adjustment for other risk factors in the Cox model (Table 3).

## Discussion

Acute GvHD is one the most frequent complications of alloHCT, reflecting alloreactivity of donor-derived T cells against mismatched minor or major histocompatibility antigens. Despite the use of prophylactic immunosuppressive protocols, based usually on calcineurin inhibitors in combination with methotrexate, it affects ~50% of alloHCT recipients (2). The most important factors influencing the risk of aGvHD are: the degree of HLA mismatch, receipt of a transplant from URD, a female donor for a male recipient and the intensity of the conditioning regimen (14, 15). While grade 1 or 2 aGvHD is usually successfully treated with topical or systemic glucocorticosteroids, its severe form is life-threatening. In particular the prognosis of patients with steroid-refractory grade 3 or 4 aGvHD is dismal with long-term OS rates not exceeding 30% (16).

In the current study we analyzed a large cohort of patients who experienced severe aGvHD after alloHCT from either MSD or URD. For the first time the analysis was focused on the impact of socioeconomic and purely economic factors at the country level on outcomes. Based on the EBMT registry, including 4,152 individuals, we clearly demonstrated that these factors are associated with TRM, PFS and OS. In contrast, factors related to organization of the transplant program at national level (team density per country area and population) as well as individual center experience had no significant impact on prognosis.

Most of the European countries belong to the well-developed group according to the Human Development Report published by the United Nations (http://hdr.undp.org/sites/default/files/hdr14-report-en-1.pdf). However, some differences exist and, as demonstrated in previous studies, values of HDI, reflecting socioeconomic status of a country correlate with the HCT rates and are associated with outcome (7, 17). In particular, as shown by the EBMT study, LFS rate for patients with acute myeloid leukemia treated in countries with the highest HDI (5th quintile) was superior compared to remaining ones (7).

The HDI is a composite index combining GDP *per capita*, life expectancy and education. Among these issues the differences regarding marcoeconomic indices in Europe appear particularly distinct. Especially, current HCE varies in a range of 465–9,720 Euro per year per inhabitant, while HCE as % of GDP is between 3.7 and 12 (Table 1). We speculated that patients with severe aGvHD may be particularly susceptible to financial issues as the treatment of this complication used to be complex and costly. The therapy is usually in-patient and requires appropriate setting. Although first line treatment consists of glucocorticosteroids and is rather cheap, it must be accompanied by supportive care including usually intravenous alimentation and anti-infectious prophylaxis. It is frequently complicated by infections requiring long-term use of expensive anti-fungal and anti-viral drugs as well as antibiotics. The treatment of steroid-refractory aGvHD is even more demanding. Attempts include e.g., the use of polyclonal antibodies, like anti-thymocyte globulin, monoclonal antibodies neutralizing pro-inflammatory cytokines, extracorporeal photopheresis or mesenchymal stromal cells (5). All these therapies are expensive themselves and may also generate secondary complications. Finally, patients who respond to initial treatment and survive, reconstitute their immune system slowly and therefore are susceptible to life-threatening infections long-term after alloHCT (18). Their incidence may be affected by the local social conditions. Early recognition of severe infections and initiation of their treatment depends on access to appropriate medical care in the area of residence but also the ability to come to the follow-up visits to transplant center.

Results of our analysis indicate that HCE strongly affects the prognosis of patients who experience severe aGvHD. In Europe current HCE is strongly correlated with HCE based on public resources, therefore, we could not distinguish between the effects of these two indices. The effect of private HCE could be demonstrated with regard to TRM and OS but associations were weaker compared to current/public HCE. Our findings correspond well with a previous EBMT study, which included adult patients with acute lymphoblastic leukemia in first remission treated with myeloablative alloHCT from MSD (9). In that study lower values of both current HCE and HDI were associated with increased risk of TRM.

Association of socioeconomic factors with the prognosis of patients with severe aGvHD has not been studied by other authors so far. Findings related to these issues were published by Karanth et al. who analyzed the incidence of grades 2–4 aGvHD and overall TRM according to recipient ethnicity (19). Among 251 patients treated in Birmingham (UK), the aGvHD rate and the incidence of TRM was higher for non-Caucasians compared to Caucasian patients. The authors speculated that the differences could be due to either differences in tumor biology or extrinsic factors such as socioeconomic factors, nutritional status, post-transplant care or presenting with late stage disease.

Hamilton et al. analyzed associations of socioeconomic factors at personal level (income, education, marital status, and work status) with outcomes of patients who developed cGvHD after alloHCT (20). Among 421 patients treated in the USA, higher income, educational level, and ability to work were associated with better quality of life while no significant associations with survival could be demonstrated.

According to results of our study the incidence of cGvHD following severe aGvHD was increased for countries with higher values of economic indices. However, cGvHD rates were analyzed as cumulative incidence with TRM representing competing risk. As TRM was lower in high income countries, the proportion of patients being at risk of developing cGvHD was higher, which could explain our findings.

It may be hypothesized that proper organization of the transplantation-related health care system may limit the negative impact of limited resources. One of its aspects is related to the number of transplant teams that should be sufficient to allow easy access for patients but also to enable sufficient center experience. Results of our study do not support its relevance with regard to the treatment of patients with severe aGvHD. Neither team density per population nor per country area were associated with outcome. As well, team activity, which was demonstrated by many studies to influence TRM had no impact on patients' prognosis in our setting (8, 21, 22).

Altogether, results of the current study indicate that macroeconomic factors, as reflected by HCE, influence strongly outcomes of patients who experience grade 3 or 4 acute GvHD following alloHCT. We postulate that our findings should be considered in interpretation of results of clinical studies in the field of aGvHD. Unfortunately, due to the retrospective nature of the study, detailed analysis of the reasons of TRM was impossible. Therefore, we could not explore in depth observed associations, which was the major study limitation. This issue should be a subject of further research. The results of such a study could guide potential actions in order to reduce mortality in countries with low HCE.

## Data Availability Statement

The datasets presented in this article are not readily available because of the EBMT regulations. Requests to access the datasets should be directed to Andrzej Frankiewicz (andrzej.frankiewicz@io.gliwice.pl).

## Ethics Statement

The study was approved by the Transplant Complications Working Party of the EBMT institutional review board and conducted in accordance with the Declaration of Helsinki and Good Clinical Practice guidelines. Since 1990, all patients provide informed consent authorizing the use of their personal information for research purposes.

## Author Contributions

All authors listed have made a substantial, direct and intellectual contribution to the work, and approved it for publication.

## Conflict of Interest

The authors declare that the research was conducted in the absence of any commercial or financial relationships that could be construed as a potential conflict of interest.
